# Supplementary material for: Identification of photoperiod-induced specific miRNAs in the adrenal glands of Sunite sheep (Ovis aries)
Source: Front Vet Sci. 2022 Jul 22;9:888207. doi: 10.3389/fvets.2022.888207 (PMC9354845; doi:10.3389/fvets.2022.888207)
Supplement: Supplementary file 1 [file Data_Sheet_1.ZIP › Supplementary file/miRDeep2 file.zip!/miRDeep2 file/Novel_Structure/SP42A1/Novel_422.pdf]

Provisional ID	:	NC_019460.2_5828
Score total	:	2.6
Score for star read(s)	:	-1.3
Score for read counts	:	0
Score for mfe	:	2.3
Score for randfold	:	1.6
Score for cons. seed	:	
Total read count	:	41
Mature read count	:	41
Loop read count	:	0
Star read count	:	0

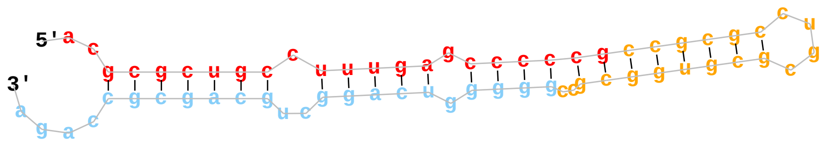

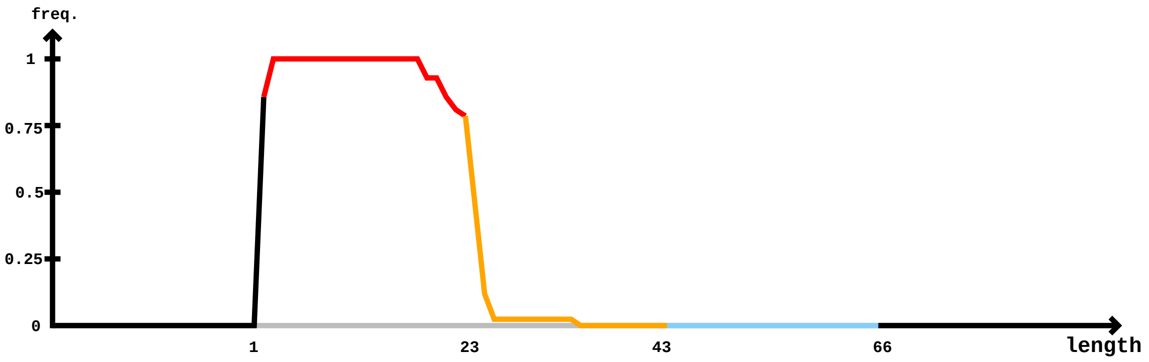

Mature Star

```
5'- ccaaucuaaaugucugaugaacgcgcugccuuugagcccccgccgcgccugcgcguggcgccgggggucaggcugcagcgccagaggacaugcucgcgcauaacugggg -3'  exp
    (((......((((((..((...(((((((.(((((.((((((((((((....))))))))..)))).)))))..)))))))))..))))))((....)).....)))..  reads  mm  sample
    ....................acgcgcugccuuugagA........................................................................  1      1   seq
    ....................acgcgcugccuuugagc........................................................................  2      0   seq
    ....................acgcgcugccuuugagccc......................................................................  2      0   seq
    ....................acgcgcugccuuugagcccc.....................................................................  1      0   seq
    ....................acgcgcugccuuugagccccc....................................................................  1      0   seq
    ....................acgcgcugccuuugagcccccg...................................................................  17     0   seq
    ....................acgcgcugccuuugagcccccgc..................................................................  8      0   seq
    ....................acgcgcugccuuugagcccccgcc.................................................................  3      0   seq
    ....................acgcgcugccuuugagcccccgccgcgccugcg........................................................  1      0   seq
    .....................cgcgcugccuuugagccc......................................................................  1      0   seq
    .....................cgcgcugccuuugagcccc.....................................................................  1      0   seq
    .....................cgcgcugccuuugagcccccg...................................................................  1      0   seq
    .....................cgcgcugccuuugagcccccgc..................................................................  2      0   seq
    .....................cgcgcugccuuugagcccccgcc.................................................................  1      0   seq
```
